# Bladder pain syndrome: validation of simple tests for diagnosis in women with chronic pelvic pain: BRaVADO study protocol

**DOI:** 10.1186/1742-4755-10-61

**Published:** 2013-12-04

**Authors:** Seema A Tirlapur, Lee Priest, Daniel Wojdyla, Khalid S Khan

**Affiliations:** 1 Women’s Health Research Unit, Barts and the London School of Medicine, Queen Mary, University of London, 58 Turner Street, London E1 2AB, UK; 2 Birmingham Clinical Trials Unit, School of Cancer Sciences, Robert Aitken Institute, University of Birmingham, Birmingham B15 2TT, UK; 3 Centro Rosarino de Estudios Perinatales (CREP), Moreno 878, Rosario S2000DKR, Santa Fe, Argentina; 4 WE1 2AB and Barts Health NHS Trust, The Royal London Hospital, Whitechapel Road, London E1 1BB, UK

**Keywords:** Bladder pain syndrome, Chronic pelvic pain, Consensus panel, Latent class analysis, Test validation

## Abstract

**Background:**

Bladder pain syndrome (BPS), a condition with no gold standard diagnosis, comprises of a cluster of signs and symptoms. Bladder filling pain and bladder wall tenderness are two basic clinical features, present in a high number of sufferers. This study will validate the performance of these simple tests for BPS in women with chronic pelvic pain (CPP).

**Methods/design:**

We will conduct a prospective test validation study amongst women with unexplained CPP presenting to gynaecology outpatient clinics. Two index tests will be performed: patient reported bladder filling pain and bladder wall tenderness on internal pelvic bimanual examination. A final diagnosis of BPS will be made by expert consensus panel. We will assess the rates of index tests in women with CPP; evaluate the correlation between index tests and Pelvic Pain Urgency/ Frequency (PUF) questionnaire results; and determine index test sensitivity and specificity using a range of analytical methods. Assuming a 50% prevalence of BPS and an 80% power approximately 152 subjects will be required exclude sensitivity of < 55% at 70% sensitivity.

**Discussion:**

The results of this test validation study will be used to identify whether a certain combination of signs and symptoms can accurately diagnose BPS.

**Trial registration:**

ISRCTN13028601

## Background

Bladder pain syndrome (BPS), formerly known as interstitial cystitis and painful bladder syndrome, is a cause of chronic pelvic pain (CPP) and is defined as CPP, bladder pressure or discomfort along with at least one other urinary symptom in the absence of any identifiable pathology or infection [1,2].

**Figure 1:**
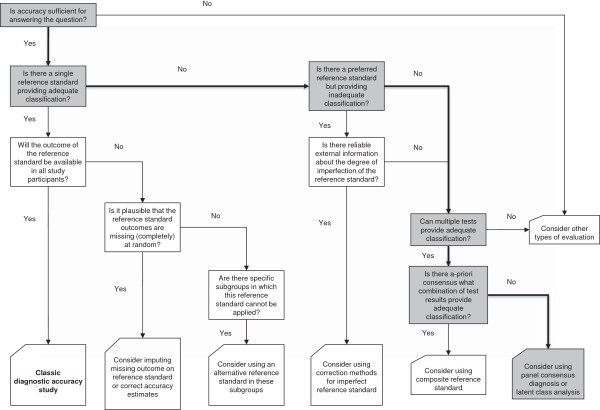
**Flow diagram showing the possible options for researchers when there is no clear reference standard in diagnostic accuracy studies [**[13]**].**

The reported prevalence of BPS is between 5 and 16 per 100,000 of the population with 61% of women presenting with CPP being diagnosed with BPS [3-5]. The condition has a large impact on sexual function and quality of life [6]. It has an unknown aetiology and imprecise characterisation, which makes it difficult to accurately diagnose clinically [7,8]. The diagnosis of BPS can be made by symptoms alone and further classified by cystoscopy findings and biopsy results, after exclusion of other confusable diseases like urinary tract infection or overactive bladder [2]. Symptoms include urinary frequency, urgency, nocturia and incomplete voiding [9]. Validated questionnaires may be used to help diagnose patients. The two commonly used are the O’Leary-Sant Interstitial Cystitis Symptom Index/Problem Index and the Pelvic Pain Urgency/ Frequency (PUF) questionnaire [10,11]. Neither questionnaire is considered a reliable predictor of disease or disease severity [11,12]. There is no gold standard test for BPS, which makes for difficulty in choice of study design for a diagnostic evaluation study (Figure 1).

The most commonly reported symptoms are bladder/pelvic pain, urgency, frequency and nocturia but this symptom cluster is present in several other urinary conditions and is not discriminating of BPS [14]. In practice, the symptom of bladder filling pain and the sign of bladder wall tenderness on vaginal examination have been shown to be present in a high number of patients with BPS, but these have not been incorporated into existing tools [15,16]. This study will validate the use of these simple tests for BPS in women with CPP.

## Methods/design

The BRaVADO study will be conducted prospectively and its protocol is reported in accordance with the SPIRIT guidelines [17]. This will be a sub-study of the MEDAL trial (MRI to Establish Diagnosis Against Laparoscopy), which is a multicentre diagnostic test accuracy study carried out in United Kingdom to investigate women with unexplained chronic pelvic pain.

Trial registration: Ethics and research and development approvals for this study are covered through the multicentre research ethics committee (REC no: 11/EM/0281). The study is sponsored by Queen Mary, University of London (Ref no: 007936 QM). Clinical trial registration no: ISRCTN13028601.

### Objectives

1. To determine the rates of the symptom of bladder filling pain and the sign of bladder wall tenderness in women with CPP.

2. To assess the correlation between bladder filling pain, bladder wall tenderness, and the PUF questionnaire (and several component questions within it) in the diagnosis of BPS in CPP.

3. To determine the prevalence of BPS in CPP, using consensus panel to establish reference standard diagnosis.

4. To estimate the accuracy with which a certain combination of signs and symptoms (index tests) can identify the diagnosis of BPS in CPP.

### Design

Prospective test validation study with consensus panel to establish reference diagnosis.

### Setting

Gynaecology outpatient clinics in the United Kingdom.

### Participant eligibility

Women presenting to secondary care with unexplained CPP. The inclusion criteria are women aged 16 or older who are referred to secondary care with unexplained CPP and have the ability to understand adequate English to give informed consent. Exclusion criteria are pregnancy, a previous hysterectomy, a proven urinary tract infection on urine dipstick and a previous diagnosis of BPS.

### Index tests

**Figure 2:**
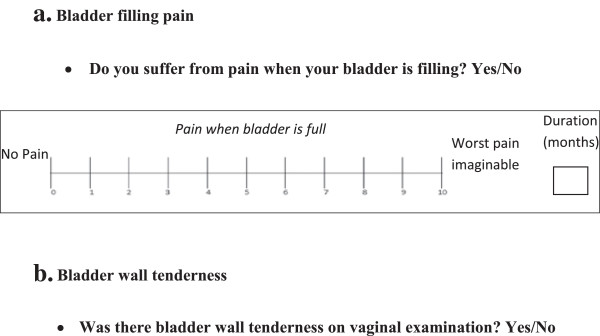
**Index test questions. a**. Bladder filling pain. **b**. Bladder wall tenderness.

1. Bladder filling pain will be assessed through a patient questionnaire (Figure 2). There is also assessment of pain when the bladder is full to discriminate the two.

2. Bladder base tenderness will be assessed by specialists in gynaecology as part of a routine vaginal examination. This is the sensation of pain when the bladder wall is palpated, rather than a sensation of discomfort.

### Reference tests

**Figure 3:**
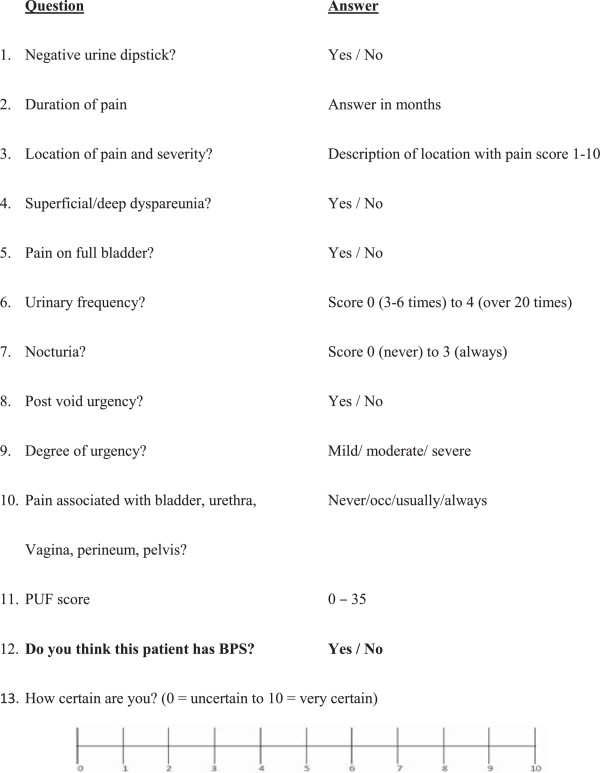
Consensus panel assessment form for symptom-based diagnosis of bladder pain syndrome.

There is no gold standard test for diagnosis. We will have an expert consensus panel in the study. The panel will be made of 3 national specialists in urogynaecology. The diagnosis determined by the panel will be a symptom-based diagnosis of BPS through patient self-reporting symptoms captured in a range of validated questionnaires. Figure 3 shows the proforma to be used for the consensus panels.

### Recruitment

**Figure 4:**
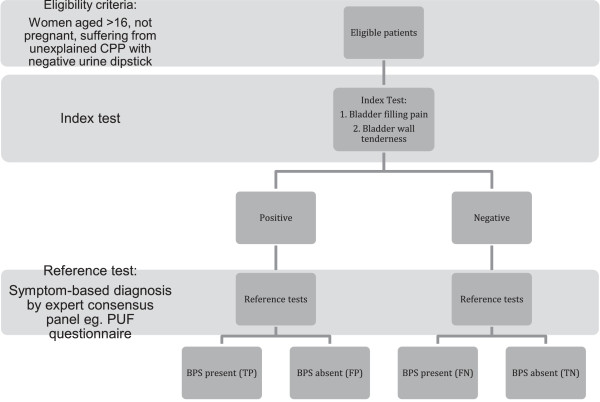
**Study flow chart in accordance with the STARD reporting guidelines [**[18]**].**

All eligible patients will be invited to participate in the study. They will be consented by named research staff at all participating centres, according to the MEDAL protocol version 1.2. There will be consecutive recruitment of all eligible patients to minimise selection bias (Figure 4).

### Sample size

The power estimation for such test validation studies is not straightforward. Estimates of prevalence of BPS vary. A recent systematic review suggested the prevalence of BPS in women with CPP is as high as 61% [3,4]. Since the exact prevalence in unknown, a range of sample sizes have been calculated based on various levels of prevalence (Table 1). There are no published estimates of sensitivity, as defined as having a positive index test and actually having BPS. We use a 95% confidence interval and exact test to estimate sample sizes, excluding a sensitivity range of less than 45% to 65% with a power of 80%. For example, assuming a 50% prevalence of BPS and an 80% power approximately 152 subjects will be required exclude sensitivity of < 55% at 70% sensitivity.

**Table 1:** Study power calculations at various assumptions

			**Sample size**		
**Sensitivity**	**Sensitivity to exclude**	**No. of patients with BPS**	**Total number of patients**		
			**40% prevalence**	**50% prevalence**	**60% prevalence**
60%	45%	82	205	164	137
65%	50%	78	195	156	130
70%	55%	76	190	152	127
75%	60%	73	183	146	122
80%	65%	69	173	132	115

### Proposed time schedule

Table 2 shows the study timeline with recruitment commencing August 2012 and study end date of September 2014 [17].

**Table 2:** **A schematic diagram showing the timeline for study participation [**[17]**]**

**TIMEPOINT**	***-t***_***1***_	**0**	***t***_***1***_	***t***_***2***_	***t***_***3***_	***t***_***4***_	***t***_***x***_
	***(July 2012)***	***(August 2012)***					
	**Pre-study**	**Enrolment**	**PatientVisit 1**	**PatientVisit 2**	**Reference diagnosis**	**Analysis**	**Study end**
**ENROLMENT:**							
**Eligibility screen**	X						
**Informed consent**		X					
**Screening log**		X					
**Urine screen**		X					
**INTERVENTIONS:**							
***Bladder filling pain***			X				
***Bladder wall tenderness***			X				
**ASSESSMENTS:**							
***Validated questionnaires***		X	X				
***Vaginal examination***			X				
***Diagnostic laparoscopy***				X			
***Expert panel (reference diagnosis)***					X		
**DATA ANALYSIS**						X	
**COMPLETE REPORT**							X

### Data collection

Data will be collected on the pre-designed data collection forms and inputted into the central database. Quality assurance testing will take place with double data entry, visual cross validation, data completeness checks and protocol adherence. All patients will undergo a diagnostic laparoscopy and cystoscopy, if deemed clinically necessary. Information will be collected about co-existing causes of CPP. The information collected will be represented in a STARD flow diagram (Figure 4).

### Data analyses

Patient characteristics will be recorded. We will provide descriptive statistics with ranges and standard deviations as appropriate. Statistical analyses will compute sensitivity, specificity and predictive values using consensus panel diagnosis as reference. We will consider several approaches to test validation [13]. The flow diagram in Figure 1 shows how we arrived at the proposed data analyses methodology. In the absence of a single reference standard to provide adequate diagnostic classification and the lack of information regarding the degree of imperfection of the reference standards, multiple tests can be used. As there is no consensus on pre-defined rules to define the target condition, we will use an expert panel diagnosis. Accuracy is concurrent criterion validity. In order to avoid incorporation bias, we will not include the index tests as part of the symptom based diagnosis. From the certainty scores of diagnosis we will calculate median and confidence interval scores, and kappa for inter-rater reliability. We will report all estimates of test performance with confidence intervals. We will also explore the use of latent class analysis, which is a statistical test that allows evaluation of a new test in the absence of a gold standard [19].

### Data monitoring

Data monitoring will be undertaken in accordance with guidelines for diagnostic studies [20]. Quality testing with range checks for data values and standard operating procedures will be used to maintain accurate data reporting and monitoring. Regular data monitoring committee meetings will be scheduled with a group of independent experts.

## Discussion

**Figure 5:**
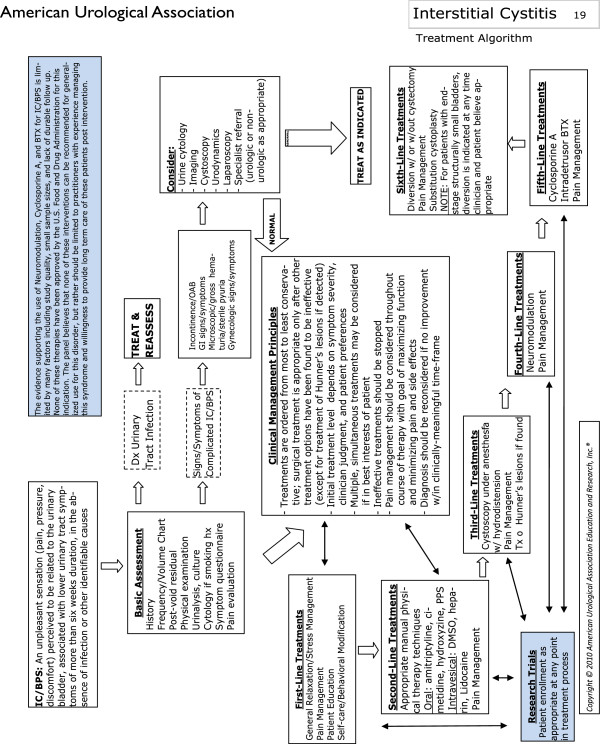
**A summary of the American urological association guidelines [**[21]**].**

The results of this test validation study will be used to identify whether a certain combination of signs and symptoms can accurately predict the diagnosis of BPS. In 2011 the American Urological Association produced their guidelines for diagnosis and management of BPS, which are summarised in Figure 5[21]. Since then, cystoscopic findings have been discredited as a negative cystoscopy does not exclude BPS and cystoscopic findings do not correlate well with disease severity or histopathology [22,23]. For this reason cystoscopy and bladder biopsy can no longer be used as a gold standard diagnostic tool for the condition. According to the 2011 guidelines, initial treatment with pain management, behavioural modifications, patient education and physical therapies can be commenced after basic assessment consisting of history, pain assessment, physical examination and urinalysis. Cystoscopy and hydrodistension are recommended as a fourth-line treatment for BPS as this investigation may provide limited diagnostic and therapeutic benefit [24]. If a cluster of signs and symptoms could accurately predict BPS this could be incorporated into the basic clinical assessment and would help clinicians diagnose the condition and initiate treatments without lengthy delays performing investigations, which are often not discriminatory.

### Ethics approval

The study has ethical approval from the National Research Ethics Service (NRES) Committee East Midlands - Nottingham 1, United Kingdom (Ref 11/EM/0281).

## Abbreviations

BPS: Bladder pain syndrome; CPP: Chronic pelvic pain; MEDAL: MRI to establish diagnosis against laparoscopy; NIHR: National institute of health research; PUF: Pelvic pain urgency/ frequency.

## Competing interests

The authors declare that they have no competing interests.

## Authors’ contributions

SAT drafted and revised the protocol and manuscript. LP revised manuscript. DW provided statistical guidance on latent class analysis and sample size calculations. KSK conceived and designed the study and applied for funding. He revised the protocol and manuscript. All authors read and approved the final manuscript.

## Author’s information

Seema Anushka Tirlapur BSc, MBChB – Clinical research fellow in obstetrics and gynaecology at Queen Mary, University of London.

Lee Priest BSc (Hons), MPhil **–** Trial Coordinator at Birmingham Clinical Trials Unit, University of Birmingham.

Daniel Wojdyla MSc – Clinical statistician, Centro Rosarino de Estudios Perinatales, Argentina.

Khalid S Khan MSc, MRCOG, MMed - Professor of women’s health and clinical epidemiology at Queen Mary, University of London.
